# Characteristics distinguishing abusive head trauma from accidental head trauma in infants with traumatic intracranial hemorrhage in Japan

**DOI:** 10.1002/ams2.341

**Published:** 2018-04-29

**Authors:** Shunsuke Amagasa, Hikoro Matsui, Satoshi Tsuji, Satoko Uematsu, Takashi Moriya, Kosaku Kinoshita

**Affiliations:** ^1^ Department of Pediatric Intensive Care Nagano Children's Hospital Azumino City Nagano Japan; ^2^ Division of Pediatric Emergency and Transport Services National Center for Child and Development Tokyo Japan; ^3^ Department of Emergency and Critical Care Medicine Saitama Medical Center Jichi Medical University Saitama Japan; ^4^ Division of Emergency and Critical Care Medicine Department of Acute Medicine Nihon University School of Medicine Tokyo Japan

**Keywords:** Abusive head trauma, diagnosis, infant, physical abuse, subdural hematoma

## Abstract

**Aim:**

To identify markers for detecting abusive head trauma (AHT) and its characteristics in the Japanese population.

**Methods:**

We retrospectively reviewed the clinical records of 166 infants with traumatic intracranial hemorrhage between 2002 and 2013 in three tertiary institutions in Japan. The infants were classified into AHT (57), suspected AHT (24), and accidental (85) group based on the defined criteria. We compared clinical presentations and computed tomography findings among these three groups and also compared age distribution of infants with AHT in our study to those in the USA.

**Results:**

Age distribution of AHT cases is significantly higher in our study than in the USA (*P* < 0.001). The rates of male sex, bruising, retinal hemorrhage, subdural hematoma, cerebral edema, and neurological sequelae were significantly higher, and those of skull fracture and scalp finding were significantly lower, in the AHT group than in the accidental group (*P* < 0.05). In the multivariable analysis of the infants with subdural hematoma, absence of skull fracture (odds ratio = 42.1; 95% confidence interval, 3.5–507.7, *P* = 0.003) was associated with AHT.

**Conclusions:**

The age range of AHT in Japan is significantly different from that of countries in Europe and North America because of familial and sociocultural situations. Absence of bruising, and rib or long bone fractures did not reduce the likelihood of AHT. Subdural hematoma without findings of an impact to the head strongly suggested AHT. Abusive head trauma is a global problem, however, diagnosis and defensive measures likely need to be tailored to accommodate cultural risk factors.

## Introduction

In cases of physical abuse, head trauma is the most common injury and is the primary cause of abuse‐related deaths. However, it is difficult to distinguish abusive head trauma (AHT) from accidental traumatic brain injury in infants, despite the fact that several systematic reviews and criteria have reported findings, such as subdural hematoma (SDH), cerebral edema/ischemia, skull fracture with intracranial injury, long bone fracture, rib fracture, retinal hemorrhage, any bruising, seizures, apnea, and inadequate history, that are associated with AHT.^1^, ^2^, ^3^, ^4^ These findings and criteria are not a gold standard for the diagnosis of child abuse. Therefore, the diagnosis of child abuse remains challenging.

Recently, the Japan Pediatric Society investigated the selected area in detail and reported that actual abuse‐related death may be three to five times more frequent than that previously reported.^5^ Child abuse is a serious social problem that should be resolved immediately. Most previous studies on AHT were from North America, Europe, and Australia. Few detailed investigations of AHT have been reported in Japan. The characteristics of AHT frequently depend on sociocultural situations surrounding the family.^6^ The characteristics and mechanisms of AHT have been largely unexplored in Japan and many cases of AHT have been overlooked.

To examine markers to help identify AHT and findings that are characteristic of AHT in the Japanese population, we investigated features that could distinguish AHT from accidental head trauma in infants.

## Methods

### Patients

We retrospectively reviewed the clinical records of 166 infants with traumatic intracranial hemorrhage detected by computed tomography (CT) scan between 2002 and 2013 in three tertiary institutions of Japan. These infants were treated for acute traumatic intracranial hemorrhage. Infants who had hemorrhagic or bone disease, or were suspected of birth injury, were excluded. Eligible infants were classified into three groups: AHT, suspected AHT, or accidental.

### Criteria for classification into AHT, suspected AHT, or accidental groups

**Figure 1:**
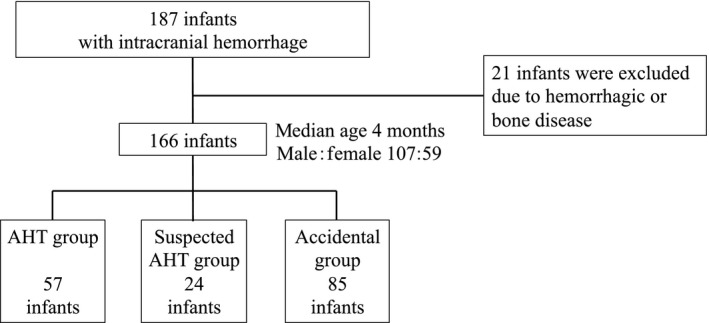
Flowchart of this population of Japanese infants with traumatic intracranial hemorrhage with abusive head trauma (AHT), suspected AHT, or accidental injury.

Methods for classifying AHT, suspected AHT, or accidental were based on the assessment of the Child Guidance Center and the criteria by Fujiwara^7^ (Fig. 1, Table 1). The infants who the Child Guidance Center suspected were victims of child abuse, and who were the focus of interventions (followed up and separated from caregiver) were classified as the AHT group. Infants not assigned to the AHT group with ≥1 of the definitional criteria were classified as the suspected AHT group. Infants who had an accident witnessed by a third party or who were not assigned to the AHT or suspected AHT group were classified as the accidental group.

**Table 1:** Determination of abusive head trauma (AHT) in infants with traumatic intracranial hemorrhage in Japan

		*n*
The Child Guidance Center suspected child abuse and intervened (followed up and separated from caregiver)		57
Definitional criteria	Total	Infants except AHT group
1) No history of trauma	28	5
2) Confession or accusation of abuse	4	0
3) Unexpected long bone fractures or old fractures, or inflicted soft tissue injury	12	0
4) SDH suggestive of AHT (bilateral, multilayer, interhemispheric, or tentorial/posterior fossa)	49	15
5) Bilateral retinal hemorrhage	32	5
Number of patients meeting the definitional criteria		
1	36	20
2	20	4
3	13	0
4	2	0
5	1	0
Total patients meeting >1 of the definitional criteria	72	24

A case with multilayer subdural hematoma (SDH) in a severe traffic accident was classified into the accidental group.

An infant who was potentially physically abused was assessed by a multidisciplinary team for child abuse prevention. This team consisted of doctors, nurses, and social workers in a hospital. When the team determined that an infant may have been abused, the team notified the Child Guidance Center (similar to Child Protective Services in the USA or UK). The Child Guidance Center comprehensively evaluated the case of AHT, and when the infant was suspected of abuse, the center intervened with the family.

Fujiwara *et al*. established the criteria based on definitions of Duhaime *et al*. and Recce *et al*.^8^, ^9^ They verified the criteria in Japan and concluded the criteria were useful for pediatricians not to condone inflicted head injury.

### Comparison among the AHT, suspected AHT, and accidental groups

We compared clinical presentations, CT findings, and outcomes between the AHT versus accidental group, and AHT and suspected AHT versus the accidental group. The clinical presentations included age, sex, initial Glasgow Coma Scale, seizures (pre‐admission), vomiting (pre‐admission), bruising other than on the head, scalp findings (scalp hematoma or head bruising), long bone or rib fractures, retinal hemorrhage, neurological sequelae, and in‐hospital death. Furthermore, we compared age distribution of AHT cases <1 year of age between the AHT group in our study and AHT cases reported in the USA.^10^ We also assessed CT findings of skull fractures, SDH and its location, epidural hematoma, subarachnoid hemorrhage, and cerebral edema.

All CT findings were based on interpretation of radiograms by a radiologist or neurosurgeon. We defined neurological sequelae as the pediatric cerebral performance category ≥2 at discharge from the intensive care unit.

### Comparison among the AHT, suspected AHT, and accidental groups in infants with SDH

As SDH is important for CT findings of AHT, we also examined only infants with SDH. We compared age, sex, and CT findings with multivariable logistic regression models.

### Statistical analysis

The χ^2^‐test was used to compare sex, clinical features, CT findings, and outcomes, and the Mann–Whitney *U*‐test was used to compare age and Glasgow Coma Scale between the AHT versus accidental group, and AHT and suspected AHT groups versus the accidental group. The Mann–Whitney *U*‐test was used to compare age between the AHT group in our study and AHT cases in the USA. The multivariable logistic regression models were used to compare the groups in SDH cases. All of the statistical tests were two‐sided, and we used an α level of 0.05 to determine significance. Data analyses were carried out using spss version 24 (IBM, New York, NY, USA).

## Results

### Comparison among the AHT, suspected AHT, and accidental groups

**Figure 2:**
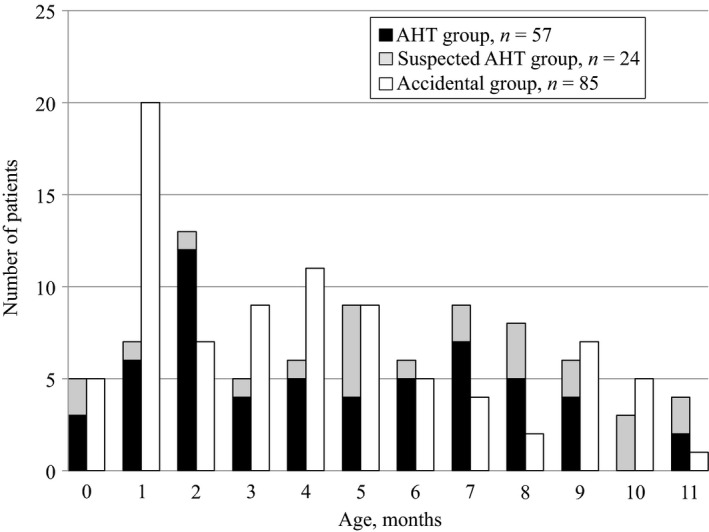
Age distribution of Japanese infants with traumatic intracranial hemorrhage classified to the abusive head trauma (AHT), suspected AHT, and accidental injury groups. Two peaks in the number of patients were observed in the AHT group, one at approximately 1–2 months and the other at 6–8 months.

**Figure 3:**
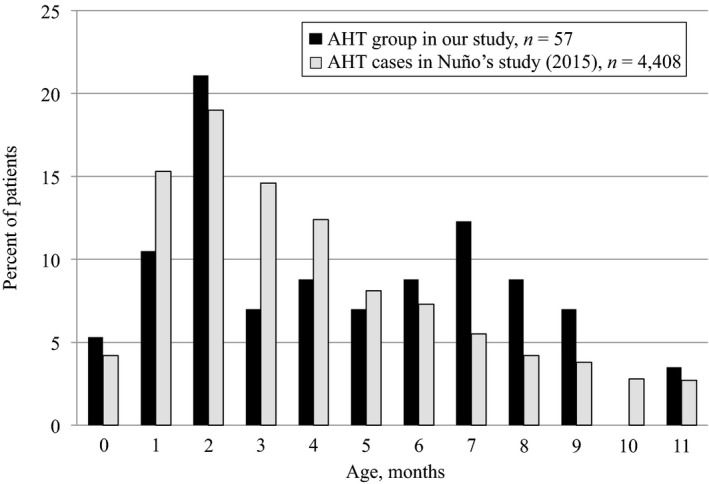
Comparison of age distribution of abusive head trauma (AHT) cases between our study and a study carried out in the USA. Age distribution of AHT cases is significantly higher in our study than in the US study (*P* < 0.001).

Among 166 infants, a total of 57 were classified into the AHT group, 24 were classified into the suspected AHT group, and 85 were classified into the accidental group (Fig. 1, Table 1). In terms of age, two peaks in the number of patients were observed in the AHT group, one at approximately 1–2 months and the other at 6–8 months (Figs 2 and 3). The age distribution of AHT cases was significantly higher in our study than in a study from the USA (*P* < 0.001; Fig. 3).

With regard to clinical presentation, the percentages of infants with bruising other than on the head (*P* = 0.004) or retinal hemorrhage (*P* < 0.001) were significantly higher in the AHT group than in the accidental group (Table 2). The percentage of infants with scalp findings was significantly lower in the AHT group than in the accidental group (*P* < 0.001).

**Table 2:** Comparison of clinical characteristics among 166 Japanese infants classified into abusive head trauma (AHT), suspected (susp) AHT, or accidental patient groups

Characteristics, *n* (%)	AHT *n* = 57	Suspected AHT *n* = 24	Accidental *n* = 85	*P*‐value	
				AHT versus accidental	AHT + susp AHT versus accidental
Age, median, months	4	6	4	0.313	0.047
Male sex	43 (74)	17 (71)	48 (57)	0.037	0.028
Initial GCS, median	11	13	15	<0.001	<0.001
Vomiting	17 (30)	8 (33)	10 (12)	0.007	0.003
Seizure	22 (39)	8 (33)	5 (6)	<0.001	<0.001
Bruising other than head	10 (18)	0	1 (1)	<0.001	0.004
Rib or long bone fracture^a^	3 (5)	0	0	0.033	0.073
Scalp findings	14 (25)	5 (21)	57 (67)	<0.001	<0.001
Retinal hemorrhage^b^	36 (63)	9 (38)	2 (2)	<0.001	<0.001
Both sides	25	8	0		
Neurological sequelae	33 (63)	8 (38)	5 (7)	<0.001	<0.001
In‐hospital death	3 (5)	1 (4)	1 (1)	0.149	0.156

a Infants who underwent skeletal survey (AHT, 53; suspected AHT, 14; accidental, 39).

b Infants who underwent fundoscopy (AHT, 55; suspected AHT, 17; accidental, 40).

GCS, Glasgow Coma Scale.

With regard to CT findings, SDH (*P* < 0.001) and cerebral edema (*P* < 0.001) were more frequently observed in the AHT group than in the accidental group (Table 3). Skull fracture (*P* < 0.001) and epidural hematoma (*P* < 0.001) were less frequently found in the AHT group than in the accidental group.

**Table 3:** Comparison of computed tomography (CT) findings among 166 Japanese infants classified into abusive head trauma (AHT), suspected (susp) AHT, or accidental patient groups

CT findings, *n* (%)	AHT *n* = 57	Suspected AHT *n* = 24	Accidental *n* = 85	*P*‐value	
				AHT versus accidental	AHT + susp AHT versus accidental
Skull fracture	13 (23)	9 (38)	62 (73)	<0.001	<0.001
SDH	51 (90)	22 (92)	22 (26)	<0.001	<0.001
Bilateral	12 (21)	4 (17)	0	<0.001	<0.001
Multilayer	10 (18)	1 (4)	1 (1)	<0.001	0.002
Interhemispheric	14 (25)	5 (21)	0	<0.001	<0.001
Tentorial/posterior fossa	4 (7)	12 (50)	0	0.013	<0.001
EDH	7 (12)	3 (13)	43 (51)	<0.001	<0.001
SAH	10 (18)	2 (8)	24 (28)	0.036	0.143
Cerebral edema	13 (23)	4 (17)	3 (4)	<0.001	<0.001

EDH, epidural hematoma; SAH, subarachnoid hemorrhage; SDH, subdural hematoma.

### Comparison among the AHT, suspected AHT, and accidental groups in infants with SDH

In the multivariable analysis of the infants with SDH, absence of skull fracture (odds ratio [OR] = 42.1; 95% confidence interval [CI], 3.5–507.7, *P* = 0.003), and SDH suggestive of AHT (OR = 259.7; 95% CI, 6.2–10881.3, *P* = 0.004) were independently associated with AHT (Table 4).

**Table 4:** Comparison of computed tomography findings in cases of infants with subdural hematoma (SDH) in multivariable logistic regression (*n* = 95)

	AHT versus accidental		AHT + susp AHT versus accidental	
	OR (95% CI)	*P*‐value	OR (95% CI)	*P*‐value
Age, months^a^	0.7 (0.9–1.0)	0.055	0.8 (0.6–1.1)	0.130
Male sex	4.1 (0.6–27.0)	0.139	2.7 (0.55–13.4)	0.222
Absence of skull fracture	42.1 (3.5–507.7)	0.003	33.2 (3.7–300.1)	0.002
Unilateral convexity	4.0 (0.2–73.6)	0.349	2.1 (0.2–21.2)	0.514
SDH suggestive of AHT	259.7 (6.2–10881.3)	0.004	331.7 (10.8–10178.0)	0.001
Cerebral edema	4.0 (0.1–10.0)	0.109	1.3 (0.2–8.8)	0.795

a A continuous predictor variable.

AHT, abusive head trauma; CI, confidence interval; OR, odds ratio; susp, suspected.

## Discussion

This study examined the clinical records of 166 infants who experienced a traumatic intracranial hemorrhage and compared the characteristics of infants who were classified as having experienced AHT or accidental head trauma in Japan. Many characteristics of AHT in our study were similar to those in previous studies from other countries. However, several novel characteristics of AHT were found in this study. First, the age distribution of AHT cases in our study is significantly higher than that reported from the USA, and the AHT group in our study had two peaks of age when there was a predilection for AHT. Second, any bruising and rib or long bone fractures were not common in cases of AHT, and there was an absence of findings of a head impact (skull fractures and findings of the scalp) in AHT. Characteristics of AHT in Japan were partially different from a previous study in countries in Europe and North America, because AHT might be affected by sociocultural status.^6^

In our study, the age distribution of AHT cases is significantly higher than that reported in the USA, and the AHT group had two age peaks for a predilection for occurrence of AHT. One peak was at approximately 1–2 months, which is consistent with previous studies,^10^, ^11^ and the other was at approximately 6–8 months. Fujiwara *et al*.^12^ also reported two age peaks for predilection of AHT in 28 cases at 2–4 months and at 7–9 months in Japan. There might be several factors to explain why older infants were inflicted in Japan. These factors could be unexpected movement, such as crawling or pulling to stand, the start of weaning food, and crying. With regard to crying, the sixth week is the peak of crying in an infant and is associated with AHT in early infancy.^11^, ^13^ Sleep‐related night‐time crying emerges by 4–24 months of age and peaks at 6–8 months in Japanese infants.^14^ Because most caregivers share a bed with newborns and infants in Japan, sleep‐related night‐time crying is often troublesome for caregivers.^15^, ^16^ A report from 2010–2011 by the Ministry of Health, Labour and Welfare in Japan described that 40–60% of cases of physical abuse‐related death in infants aged 1–11 months resulted from intolerance to crying. These findings suggest that intense crying, such as sleep‐related night‐time crying, and sleep sharing could be possible factors for abuse in older infants. Further research is required to clarify the causes associated with abuse in the 6–8 month age range in Japan.

Bruising other than on the head, and long bone or rib fractures were only observed in 18% and 5% of the AHT group, respectively, in our study. Previous studies from countries in Europe and North America reported that bruising other than on the head and long bone or rib fractures were observed in 37–46% and 23–51% of AHT cases, respectively.^9^, ^17^, ^18^, ^19^ While a previous study in Japan reported that an impact is a common mechanism of AHT and external findings are more common than those in the USA,^20^ these conceptions may mislead to overlooking of AHT in Japan. In the current study, long bone or rib fractures and bruising other than on the head had a high specificity but a low sensitivity for the diagnosis of AHT.

In this study, findings of impact on the head were significantly less frequent in the AHT group than in the accidental group. An absence of findings of an impact to the head, especially in cases of SDH, was strongly associated with AHT. Although skull fracture, head bruising, and scalp hematomas are evidence of impact to the head, absence of these findings does not mean an absence of impact. Impact against a soft surface may dissipate the impact forces over a larger area, leading to rapid deceleration without skull fracture. This has been shown to result in forces that are many times higher than those observed with shaking. Because AHT consists of various movements, such as shaking, an impact, and primary or secondary brain injury, medical professionals should work to better recognize the signs and symptoms of abusive‐related head injury, including those caused by both shaking and blunt impacts.^21^

Our study has several limitations. First, we retrospectively classified the patients as AHT, suspected AHT, or accidental and there may have been some misclassification. Skeletal surveys or fundoscopies were not completed in some cases. Moreover, the ophthalmology consultation did not always occur within 24 h, and subtle retinal hemorrhages might disappear. Second, circulatory bias occurs in this study, as with many studies on AHT. Lynøe *et al*.^22^ reported that, because of problems associated with classifying AHT, and circular reasoning, there was insufficient evidence on which to assess the diagnostic accuracy of the triad (SDH, retinal hemorrhage, and encephalopathy) while attempting identify traumatic shaking. To reduce misclassification and circulatory bias, we adopted the multidisciplinary decision‐making capacity of the Child Guidance Center. The percentage of families that received notification to and/or interventions by the Child Guidance Center was lower in Japan than in other countries as a result of a rapid increase of reported child abuse.^12^, ^23^ The rate of AHT group in this study was lower than that (53–60%) in previous studies of Europe and the US.^18^, ^24^, ^25^ The children who were the focus of Child Guidance Center interventions might have been at higher risk for AHT. However, because some items which were used in the comparison of this study included information for making a decision of abuse, circularity bias may occur. The bias should be acknowledged and further prospective analyses should be taken with much caution to avoid this type of bias when classifying AHT and accidental head trauma. Finally, this study included data from only three institutions over a limited period. However, our study is the largest multicenter study of AHT in Japan and provided important preliminary findings.

## Conclusion

The age range of AHT in Japan is significantly different from that of countries in Europe and North America because of familial and sociocultural situations. Absence of bruising and rib or long bone fractures did not reduce the likelihood of AHT. Subdural hematoma without findings of a direct impact to the head strongly suggested AHT. Abusive head trauma is a global problem; however, diagnosis and interventions for prevention of AHT will likely need to be tailored to accommodate cultural risk factors. Moreover, circularity bias should be acknowledged and further prospective analyses should be carried out with much caution.

## Disclosure

Approval of the research protocol: The Ethical Board of Nagano Children's Hospital approved this retrospective study (Receipt number 27‐39).

Informed consent (if applicable): N/A.

Registry and the registration no. of the study/trial: N/A.

Animal studies (if applicable): N/A.

Conflict of interest: None declared.
